# A chimeric HS4 insulator–scaffold attachment region enhances transgene expression in transfected Chinese hamster ovary cells

**DOI:** 10.1002/2211-5463.12335

**Published:** 2017-11-06

**Authors:** Si‐jia Chen, Wen Wang, Feng‐yi Zhang, Yan‐long Jia, Xiao‐yin Wang, Xiao Guo, Shao‐Nan Chen, Jian‐hui Gao, Tian‐Yun Wang

**Affiliations:** ^1^ Department of Biochemistry and Molecular Biology Xinxiang Medical University Henan China; ^2^ Pharmacy Collage Xinxiang Medical University Henan China; ^3^ Henan Collaborative Innovation Center of Molecular Diagnosis and Laboratory Medicine Xinxiang Medical University China; ^4^ Grade 2012 The Third Clinical Medical College of Xinxiang Medical University Henan China

**Keywords:** characteristic motif, Chinese hamster ovary, enhanced green fluorescent protein, hypersensitive site 4–scaffold attachment region, scaffold/matrix attachment regions, transgene expression

## Abstract

Chinese hamster ovary (CHO) cells are one of the most commonly used expression systems for the production of recombinant proteins but low levels of transgene expression and transgene silencing are frequently encountered. Epigenetic regulatory elements such as the chicken β‐globin locus control region hypersensitive site 4 (HS4) and scaffold/matrix attachment regions (S/MARs) have positive effects on transgene expression. In this study, a chimeric HS4‐SAR was cloned upstream or downstream of an enhanced green fluorescent protein (eGFP) expression cassette in a eukaryotic vector, and the resulting vectors were transfected into CHO cells. eGFP was detected by flow cytometry. Real‐time quantitative PCR (qPCR) was used to determine copy numbers of the stably transfected cells. And fluorescence *in situ* hybridization (FISH) was used to detect the status of vector in the host cell chromosome. The results showed that HS4‐SAR positioned downstream of the expression cassette could enhance eGFP expression by 4.83‐fold compared with the control vector. There may not be a relationship between transgene copy number and gene expression level. HS4‐SAR did not appear to alter the integration of the transgene into the host cell chromosome or its position in the chromosome. We found a synthetic chimeric HS4‐SAR positively increased transgene expression in CHO cells.

**Abbreviations**
CHO: Chinese hamster ovary
eGFP: enhanced green fluorescent protein
FISH: fluorescence *in situ* hybridization
GAPDH: glyceraldehyde phosphate dehydrogenase
HS4‐SAR: hypersensitive site 4–scaffold attachment region
S/MARs: scaffold/matrix attachment regions

Since genetic engineering techniques were first developed in the 1970s, molecular biology technology has advanced rapidly. The Chinese hamster ovary (CHO) cells expression system is one of the most commonly used expression systems for the production of recombinant proteins, which has many advantages, including precise post‐transcriptional modification function, production of proteins resembling native proteins in terms of molecular structure, high efficiency of recombinant gene amplification and expression, stable integration of exogenous genes into the CHO cell chromosome, and the ability to be cultured under adherent or suspension conditions 1, 2, 3. However, some limitations leading to low levels of transgene expression and transgene silencing have restricted the wide use of the CHO cell expression system 4, 5.

Scaffold/matrix attachment regions (S/MARs) can block transgene silencing 6, 7, 8 and increase transgene expression levels and stability in host cells 9, 10, 11, 12, 13, 14, 15. In addition, S/MARs can also reduce variations in transgene expression among different cells to some extent, and the rate of transgene genomic integration can be increased 11. However, some reports have shown that S/MARs have inconsistent effects on transgene expression 16, 17, 18.

Scaffold/matrix attachment regions play important roles in defining the structural units of chromatin, functioning as boundary elements bordering the regions of a condensed or open chromatin structure 19. S/MARs are special DNA sequences that exist in chromatin of eukaryotic cells and can combine with the nuclear matrix. S/MARs are AT‐rich sequences that are ~ 300–2000 bp in length and contain an A‐box, T‐box, *Drosophila* topoisomerase II recognition sites, and curved DNA. The secondary structure of MARs contains narrow DNA and the small groove, making the chain easy to curve and melt. Insulators are *cis*‐acting regulatory sequences that enhance blocking activity to prevent the spread of heterochromatin and silencing of genes 20. The chicken hypersensitive site 4 (cHS4) is one of most commonly used and best characterized insulators and possesses both enhancer‐blocking and barrier activity 21, 22. Some reports have described the effects of cHS4 and MARs on transgene expression; however, how the combination of the two elements elevates transgene expression is unclear. In a previous study, a chimeric hypersensitive site 4–scaffold attachment region (HS4‐SAR) insulator was shown to prevent silencing and enhance the expression of lentiviral vectors in pluripotent stem cells 9. Whether this sequence can affect transgene expression in CHO cells has not been evaluated.

In this study, a chimeric HS4‐SAR was synthesized and ligated to the upstream or downstream region of expression cassettes in a eukaryotic vector, and transfected into CHO cells, and further studied the effects and mechanism of the chimeric HS4‐SAR on transgene expression in stably transfected CHO cells.

## Materials and methods

### HS4‐SAR synthesis and vector construction

According to a previously reported sequence 9, an HS4‐SAR DNA fragment was synthesized by General Biosystems (Chuzhou, China). The synthetic HS4‐SAR DNA fragment was cloned into upstream or downstream region of the expression cassette of pIRES‐eGFP, which was obtained via cloning the enhanced green fluorescent protein (eGFP) from peGFP‐C1 (Clontech, New York, NY, USA) into the pIRES‐neo vector (Clontech). The synthetic MAR was ligated with pIRES‐eGFP. All procedures were performed according to the standard methods 23.

### Cell culture and transfection

CHO‐S cells (Life Technologies # A11557‐01; Thermo Fisher Scientific, New York, NY, USA) were plated at a density of 2 × 10^5^ cells per well in 24‐well plates. The cells were cultured in protein‐free, serum‐free, chemically defined CD CHO medium (Life Technologies # 10743‐029) supplemented with 8 mm l‐glutamine (Life Technologies # 25030‐024) in 125‐mL Corning shake flasks (Sigma # 431255; San Francisco, CA, USA) with 30 mL medium in a humidified incubator at 37 °C with 5% CO_2_. On the second day, after reaching 80% confluence, the cells in each well were transfected with the pIRES‐sMAR3, pIRES‐MAR5, and pIRES‐eGFP vectors using 1 μL Lipofectamine 3000 Transfection Reagent per μg vector (Invitrogen, Carlsbad, CA, USA) according to the manufacturer's instructions. At 48 h post‐transfection, G418 (800 μg·mL^−1^) was added to screen the transfected cell lines.

### Transient expression

At 48 h post‐transfection, the transfection efficiency and transient eGFP expression levels were analyzed by evaluating the fluorescence intensity in transfected cells by fluorescence microscopy (Nikon ECLIPSE Ti, Nikon, Japan). For visualizing the cells clearly, in this study we set fluorescence microscopy as follows: The cells were magnified 200 folds. Moreover, the fluorescence microscopy can acquire an emission wavelength of 530 nm using a 530/15 bandpass filter for the green fluorescence. The cells transfected with different vectors were collected to detect the eGFP‐positive cells and mean fluorescence intensity (MFI) by flow cytometry.

### Screening stability of transfected cells and flow cytometry analysis

Stably transfected cell lines were selected using G418 (800 μg·mL^−1^) at 48 h after transfection. Approximately 2 weeks after transfection, stable transfected cell colonies formed, and the cells were cultured with G418 (500 μg·mL^−1^). At 20 days post‐transfection, when the cells reached 90% confluence, we collected the cells and analyzed the expression of eGFP by flow cytometry. eGFP expression levels were determined by measuring the MFI.

### Real‐time quantitative PCR

To assess the relationship between eGFP expression and gene copy number, the cells were collected 30 days after transfection, and genomic DNA was extracted for analysis by quantitative PCR (qPCR). Primers were designed according to the sequence of *eGFP*, as follows: F1, 5′‐CTACGTCCAGGAGCGCACCATCT‐3′ and R1, 5′‐GTTCTTCTGCTTGTCGGCCATGATAT‐3′. The glyceraldehyde phosphate dehydrogenase (*GAPDH*) gene was used as an internal reference, and the primer sequences were designed as follows: F1, 5′‐CGACCCCTTCATTGACCTC‐3′ and R1, 5′‐CTCCACGACATACTCAGCACC‐3′.

Before qPCR, the DNA for all samples was adjusted to the same concentration using deionized water. qPCR was carried out in a final volume of 10 μL containing 4 μL template DNA (0.05 μg·μL^−1^), 5 μL SYBR Green, 0.2 μL of each of the forward and reverse primers (10 μm each), and 0.6 μL deionized water. The PCR protocol was as follows: 95 °C for 3 min; 30 cycles of 94 °C for 30 s, 50 °C for 30 s, and 72 °C for 30 s; and 60 °C for 5 min. All samples were evaluated three times. Through qPCR, the *C*_t_ value can be obtained. Moreover, relative eGFP copy numbers were calculated by the $2^{-\Delta \Delta C_{t}}$ method.

### Fluorescence *in situ* hybridization (FISH) analysis

The cells were cultured and passaged in medium containing G418 (500 μg·mL^−1^). At 30 days post‐transfection, the cells were collected for fluorescence *in situ* hybridization (FISH) analysis. The number of fluorescent probes and the presence of the vector in the chromosomes of CHO cells were observed under a fluorescence microscope.

### Statistical analysis

All data were obtained from at least three independent experiments and were analyzed using spss 18.0 software (SPSS Inc., Chicago, IL, USA). Data are reported as means ±standard deviations. Comparisons between different groups were analyzed using single factor ANOVA, and *t*‐tests were performed for pairwise comparisons. Differences with *P* values of < 0.05 were considered statistically significant.

## Results

### Characteristics of the HS4‐SAR sequence

**Figure 1:**
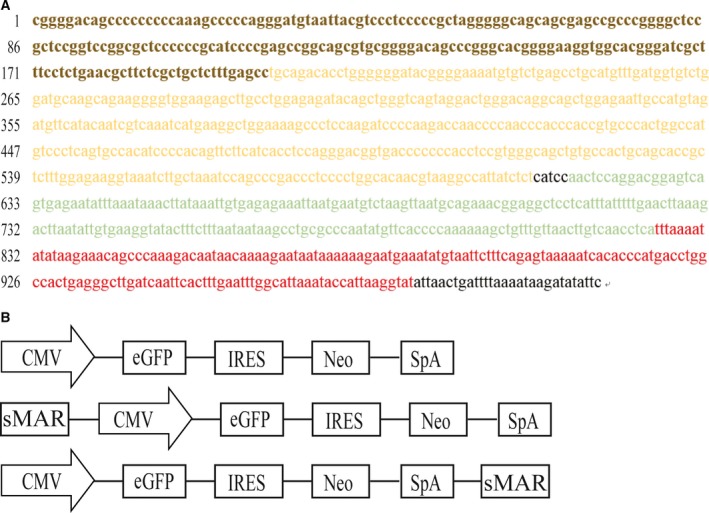
Synthesis of chimeric HS4‐SAR sequence and plasmid construction. According to reported chimeric HS4‐SAR sequence, HS4‐SAR was synthesized. The yellow (both light and dark) represents HS4 insulator, and the dark yellow is core sequence of HS4 insulator. The green represents interferon‐beta matrix association region. The red represents immunoglobulin matrix association region (A). The synthesis chimeric HS4‐SAR sequence was inserted into the upstream or downstream region of an enhanced green fluorescent protein (eGFP) expression cassette in pIRES‐eGFP to construct the pIRES‐sMAR5 and pIRES‐sMAR3, respectively (B). CMV, cytomegalovirus major immediate early; eGFP, enhanced green fluorescent protein; IRES, internal ribosome entry site; sMAR, synthetic matrix attachment region; SpA, simian virus 40 early polyadenylation signal.

The HS4‐SAR sequence was synthesized according to a previous study (Fig. 1A). The sequence contained the HS4 insulator sequence, binding sites, interferon‐beta matrix association region, and immunoglobulin matrix association region. The synthetic MAR was inserted into the upstream or downstream region of expression cassettes in the pIRES‐eGFP vector. New vectors were constructed (pIRES‐sMAR3 and pIRES‐sMAR5; Fig. 1B), and the pIRES‐eGFP vector was used as a control.

### Analysis of transfection efficiency and transient expression

**Figure 2:**
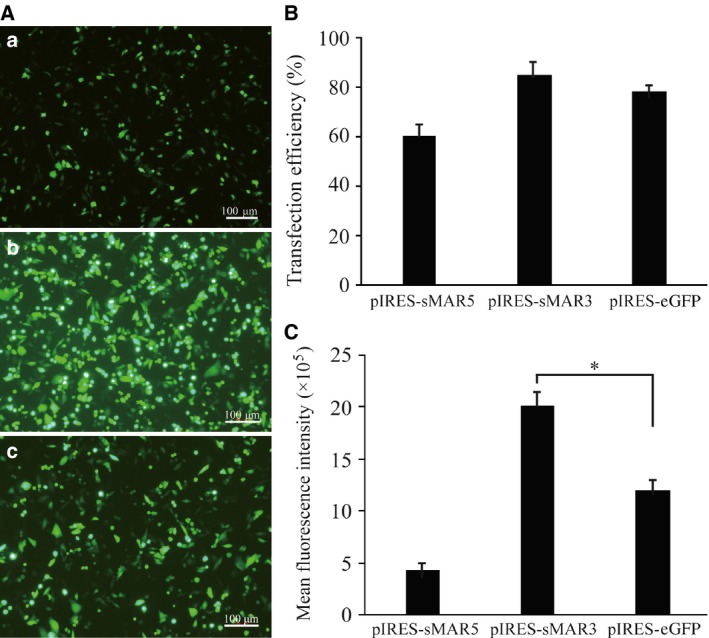
Fluorescence microscopy of eGFP gene in transfected CHO cells after 48 h of transfection. (A) Micrograph of cells transfected with pIRES‐sMAR5 vectors (a), pIRES‐sMAR3 (b) vectors, and pIRES‐eGFP vectors (c). (B) Cell transfection efficiency was detected using eGFP antibody by flow cytometry. (C) Meanwhile, the transient expression levels of eGFP were obtained (*P <* *0.05).

At 48 h after transfection, the fluorescence intensity was observed using a fluorescent microscope (Fig. 2A). Meanwhile, the cells were collected to detect the transfection efficiency and MFI using flow cytometry. The results showed that the transfection efficiency of pIRES‐sMAR3 was significantly higher than that of pIRES‐eGFP (Fig. 2B). In the meantime, the fluorescence intensity of cells transfected with the pIRES‐sMAR3 vector was higher than that of cells transfected with the pIRES‐eGFP (2.01 × 10^6^±0.47 × 10^3^ versus 1.2 × 10^6^ ± 0.24 × 10^3^) vector. In contrast, the MFI of cells transfected with the pIRES‐sMAR5 (4.3 × 10^5^ ± 0.10 × 10^3^) vector was lower than that of cells transfected with the control vector (Fig. 2C). Thus, HS4‐SAR increased transgene expression when the synthetic HS4‐SAR was inserted into the expression cassette at the downstream region. However, when the synthetic HS4‐SAR was inserted into the upstream region, the transgene expression level was not increased.

### Analysis of stably expression

**Figure 3:**
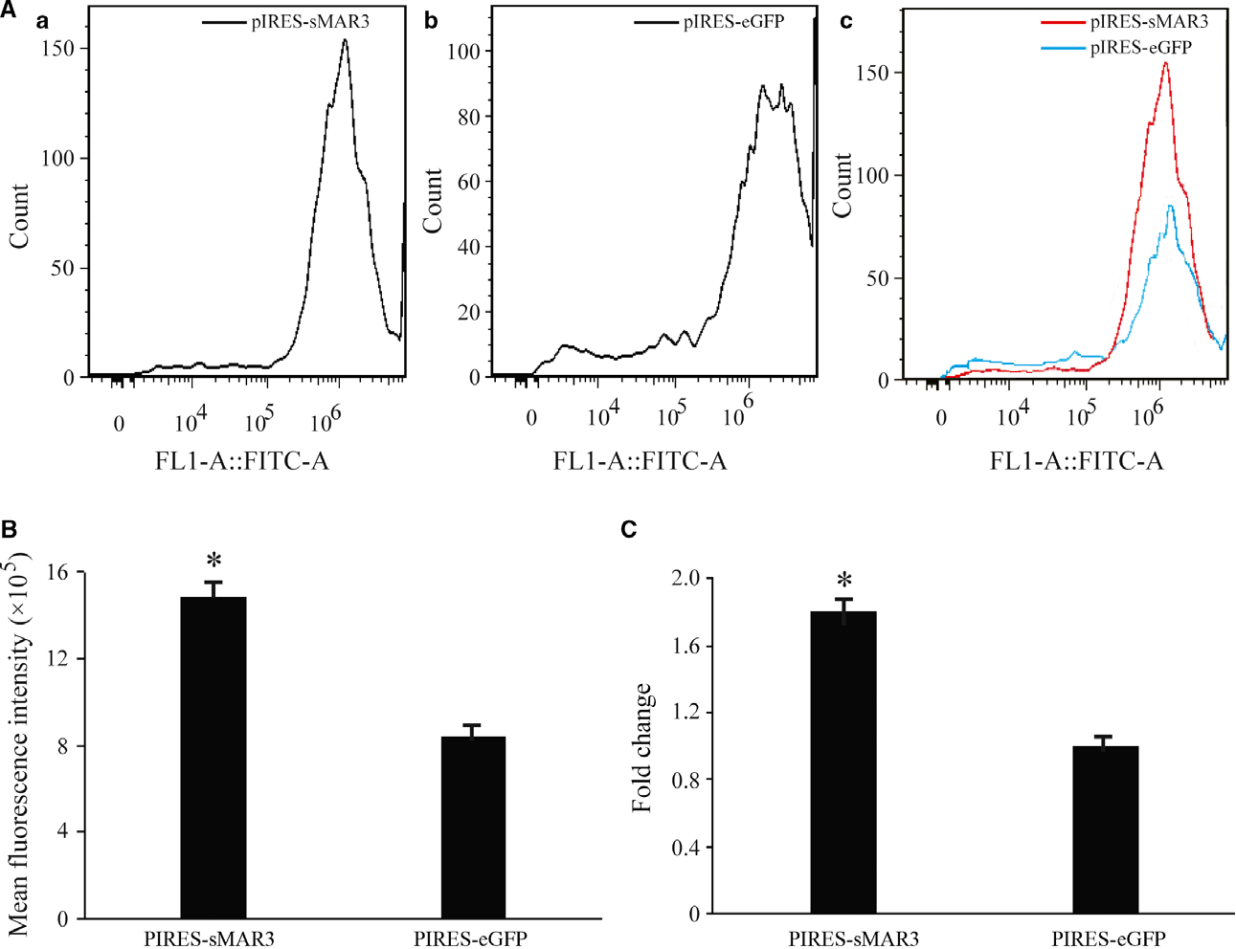
Analysis of the stable transgene eGFP expression. Cells were collected under G418 screening at day 20 post‐transfection. (A) Micrograph of cells that express different eGFP expression levels and transfected with pIRES‐sMAR3 vector (a) and pIRES‐eGFP vector (b). And the cell counts at different eGFP expression levels were compared (c). B) Flow cytometric analysis of transgene eGFP expression. After 20 days of transfecting vectors, recombinant protein expression stability was tested using flow cytometric analysis. The eGFP expression level was represented by the MFI. (C) The fold change of eGFP expression levels in cells transfected with pIRES‐sMAR3 and pIRES‐eGFP vectors was calculated (*P <* *0.05).

At 48 h after transfection, G418 (800 μg·mL^−1^) was used to screen the cells transfected with vectors. When the cells untransfected with vector were killed, the stably transfected cell colony appeared. Then, we observed the fluorescence intensity using a fluorescent microscope. Additionally, we collected stably transfected CHO cells and measured the fluorescence intensity using a flow cytometry (Fig. 3A). The mean MFI of the cells transfected with pIRES‐sMAR3 (1.41 × 10^6^ ± 8.9 × 10^3^) was higher than that of the cells transfected with control (8.39 × 10^5^ ± 1.7 × 10^3^; Fig. 3B); the fold change of sMAR downstream expression cassette of the vector on transgene expression levels was calculated (Fig. 3C). The highest of eGPF gene expression in the pIRES‐sMARs was 4.83‐fold compared with control vector. That suggests that HS4‐SAR could enhance transgene expression when inserted into the downstream region of the expression cassette of the vector in stably transfected CHO cells.

### Analysis of long‐term transgene expression stability

**Figure 4:**
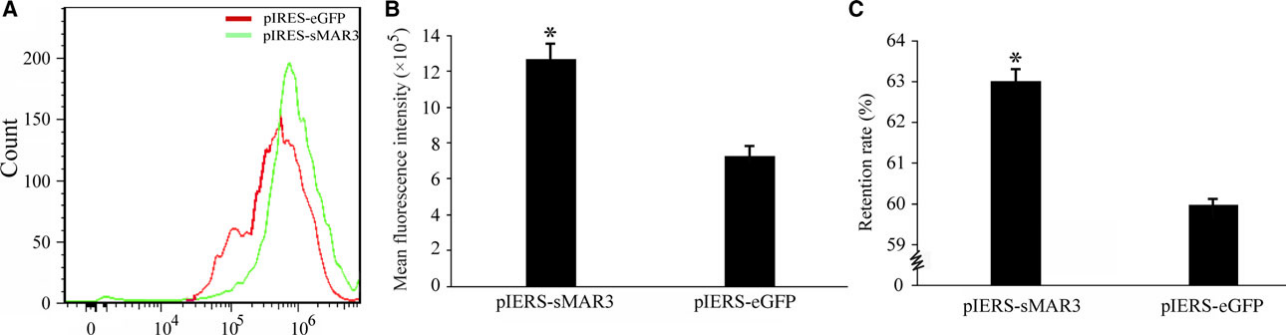
Evaluation of stability of long‐term transgene expression. Stably transfected CHO cells were cultured in G418 (500 μg·mL^−1^), and the cells were collected again and MFI by flow cytometry at days 90 after transfection. (A) Flow cytometric analysis of transgene eGFP expression levels. (B) The eGFP expression level was represented by the MFI. (C) Statistical analysis of recombinant protein expression rate (*P <* *0.05).

The stability of transgene expression is an intractable problem in the production of recombinant proteins. At 90 days after transfection, the cells were collected again, and the fluorescence intensity was measured using flow cytometry (Fig. 4A). The fluorescence intensities of cells transfected with pIRES‐sMAR3 and pIRES‐eGFP were 1.27 × 10^6^ ± 1.3 × 10^3^ and 7.28× 10^5^ ± 0.64 × 10^3^, respectively (Fig. 4B), demonstrating that the synthetic HS4‐SAR could improve transgene expression. Additionally, the retention rates of synthetic HS4‐SAR and the control were 63.0% and 60.0%, respectively, compared with the fluorescence intensity at 20 days (Fig. 4C). Accordingly, we concluded that synthetic HS4‐SAR enhanced the stability of increased transgene expression.

### Transgene copy number analysis

**Figure 5:**
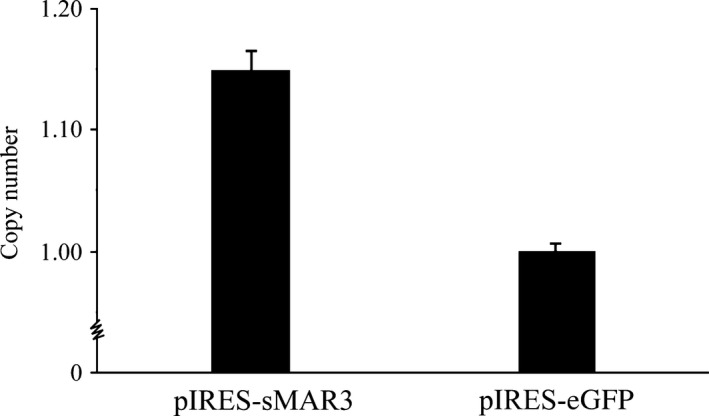
Gene copy number was determined with fluorescent quantitative PCR. We collected the transfected cells that were cultured in G418 (500 μg·mL^−1^) at 30 days post‐transfection. The copy numbers were tested using fluorescent quantitative PCR. And copy number's mean values differed between the vectors containing the HS4‐SAR and control (*P *<* *0.05).

Whether there is a relationship between gene copy number and transgene expression is unclear. Therefore, we next analyzed the gene copy numbers in cells transfected with the above‐mentioned vectors. The mean relative copy number of the pIRES‐sMAR3 vector was 1.15 ± 0.32, as determined by setting the copy number of the control vector to 1 (Fig. 5). Combined with our previous analysis of eGFP expression levels, these data suggested that there may not be a relationship between transgene copy number and gene expression level.

### FISH analysis

**Figure 6:**
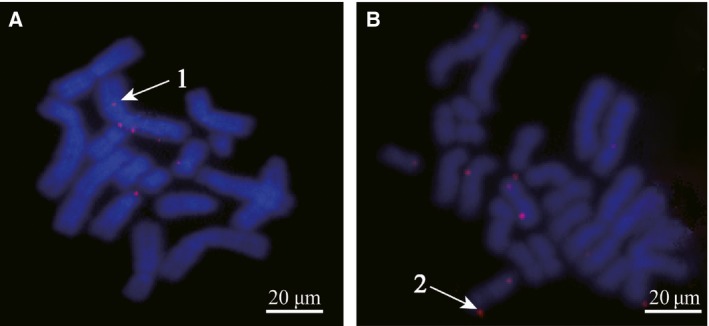
The status of plasmids in transfected CHO cells. At 30 days post‐transfection, the cells cultured in G418 (500 μg·mL^−1^) were collected and tested by FISH analysis. pIRES‐sMAR3 (A), pIRES‐eGFP (B). 1: episomal; 2: integrated.

Some reports demonstrated that integrated vectors can improve transgene expression stability. To detect the state of the vector in the CHO cell chromosome, we performed FISH analysis on spread chromosomes from CHO cells transfected with pIRES‐sMAR3 and pIRES‐eGFP vectors at 30 days after transfection in the presence of G418 selection pressure. Five metaphase plates were analyzed by FISH for each vector. FISH analysis showed that the vector has two forms in the host cell chromosome, episomal and integrated. The status of transgene in the chromosome mediated by MAR element showed no significant difference, and transgenes did not appear to be targeted to specific chromosomal locations (Fig. 6A,B).

## Discussion

A recombinant protein produced by a mammalian expression system has many advantages, including strong specificity, low toxicity, few side effects, and clear biological function, compared with micromolecules making up chemical medicines. The CHO cell system is an important mammalian expression system 24, 25. However, owing to epigenetic effects, for example, silencing of transgenes, low efficiency, and unstable transgene expression limit the wide application of the CHO cell system for the production of recombinant proteins 26, 27.

Matrix attachment regions can increase expression levels of the transgene in stably transfected CHO cells 6, 7, 8, 9, 10, 11, 12. However, the characteristics and mechanism of MARs function have not been elucidated, and further studies are needed to develop improved methods for transgene expression. In our study, a synthetic HS4‐SAR sequence (1010 bp in length) was designed. The results indicated that insertion of the HS4‐SAR sequence into the 3′‐end of the pIRES‐eGFP vector could increase transgene expression. However, insertion of the MAR sequence into the 5′‐end of the vector did not increase transgene expression. The position of MAR in the vector can affect the transgene expression levels. MAR can increase transgene expression in CHO cells when inserted upstream of the promoter and enhancer 28, 29, 30. However, it has been demonstrated that MAR's enhancing effect was significant when downstream of the transgene and poly A 31. This may be that MAR acts boundary elements, but the function needs the synergistic effect of insulator. If MAR confers the enhancer's effect, MAR can increase transgene expression, not the position effect. Other MARs did not have the enhancer's function, which increases transgene expression only through boundary elements. They can function when MARs are separated by insulator (polyA) elements downstream of the expression cassette.

There are two forms of expression vector on the chromosome of host cell, episomal and integration. Girod *et al*. 29 found that MAR element did not reveal a high occurrence of multiple integration events or of abnormal chromosomal structures. In the present study, the FISH results showed that the status of pIRES‐sMAR3 and pIRES‐eGFP vectors was not significantly different; the episomal and integration statuses exist in MAR‐containing vector and the control. This result indicated that MAR's enhancing effect had no influence on the status of vector in the chromosome of the host cells, which is consistent with the previous study 29.

In a previous study, X‐29 and 1–68 were found to be optimal MAR sequences for improving transgene expression 29. X‐29 and 1–68 were 3492 and 3630 bp in length, respectively. Some epigenetic regulators, such as special (A + T)‐rich binding protein 1 (SATB1), nuclear matrix protein 4 (NMP4), and CCCTC‐binding factor (CTCF), can be bound by MARs 32, 33. X‐29 and 1–68 contain the characteristic motifs of A‐box, T‐box, Hox, CCAAT enhancer‐binding protein (CEBP), NMP4, and forkhead activin signal transducer‐1 (FAST1). Compared with X‐29 and 1–68, the synthetic MAR sequence contains the characteristic motifs of A‐box, T‐box, topoisomerase II, CTCF, upstream regulatory factor (USF), as well as the HS4 insulator sequence, interferon‐beta matrix association region, and immunoglobulin matrix association region. CTCF and USF may bind with the HS4 insulator sequence, interferon‐beta matrix association region, and immunoglobulin matrix association region to enhance transgene expression through MARs. MAR's function can be predicted; more potent MARs can be used to improve recombinant protein production through analysis of the MAR characteristic motif 29, 34. The stable expression of a transgene requires that the vectors are integrated into the chromosome of host cells 35, 36. The source of MARs, the inserted position of MARs, and the type of host cells may affect transgene expression by MAR 5, 30, 37, 38. Moreover, gene copy numbers are related to transgene expression 39, and the methylation of DNA may reduce transgene expression 40, 41, 42. Some reports have demonstrated that MARs improve the expression of transgenes through the recombination pathway of synthesis‐dependent microhomology‐mediated end‐joining (MMEJ) 8, 43, 44. However, none of these studies assessed the effects of the characteristic sequences of MARs on transgene expression. Only by elucidation of the structure and mechanisms of MARs can we better resolve the epigenetic effects of transgene expression. In this study, the synthetic HS4‐SAR was inserted into different positions in the vector to evaluate the effects of position, characteristic motifs, and copy numbers on transgene expression. Detection of eGFP expression by flow cytometry demonstrated that MARs could increase transgene expression, and FISH analysis showed that the vector has two forms in the host cell chromosome, episomal and integrated.

The reporter gene was used as the target gene, and no therapeutic proteins were studied. The synthetic HS4‐SAR can increase transgene expression level; however, whether HS4‐SAR can function in other expression systems needs to be explored. In summary, we found that HS4‐SAR could effectively increase and maintain transgene expression when inserted downstream of the transgene and poly A, and the effect may not be caused by transgene copy numbers increasing and the status of vector in the chromosome of the host cells. In the following studies, the gene of interest for recombinant protein production and mechanisms underlying these effects will be investigated and elucidated.

## Author contributions

T‐YW designed and analyzed the experiments and wrote the manuscript. S‐JC, WW, and Y‐LJ performed the experiments and co‐wrote the manuscript. XG designed and analyzed experiments for Figs 2 and 3 and co‐wrote the manuscript. S‐NC and F‐YZ performed the experiment for vector construction and cultured cells. X‐YW and J‐HG co‐revised the manuscript and helped in statistical analysis.
